# How the methodology determines the outcome of the in vitro micronucleus assay (OECD TG 487): a comparison of the MicroFlow and the microscopic evaluation approach highlights the impact of cytotoxicity/cytostasis metrics in V79 cells for matrine

**DOI:** 10.1007/s00204-025-04271-1

**Published:** 2026-01-14

**Authors:** Benjamin Christian Fischer, Yemurai Musengi, Kristin Herrmann, Carsten Kneuer, Jeannette König

**Affiliations:** Department Pesticides Safety, German Federal Institute for Risk Assessment, 10589 Berlin, Germany

**Keywords:** Micronucleus, MicroFlow, Cytotoxicity, Cytostasis, RICC, RPD, CBPI, Matrine, OECD TG 487

## Abstract

The in vitro micronucleus (MN) test (OECD TG 487) has become the gold standard in various regulatory silos for assessing compounds for their potential to cause clastogenicity. Its main advantages are the reproducibility and robustness, as well as the ability to investigate both structural and numerical chromosome damage, i.e. clastogenicity and aneuploidy. A disadvantage, however, can be seen in the resource-intensive microscopical evaluation. Consequently, faster, high-throughput methods, such as flow cytometry have been developed and integrated into the test guideline protocol. Regardless of the evaluation method applied, results must be consistent to justify the mutual acceptance of data established for OECD TG tests performed under GLP. This may be particularly challenging for substances of lower potency. Here, we present two case studies comparing the results of the MN test according to TG 487 with microscopic evaluation with those obtained using the MicroFlow kit (Litron Laboratories). The MN tests were performed with V79 cells without cytochalasin B, incubated for 4 h (with and without metabolic activation) or 24 h with 0.5, 1.0, 1.25, 1.375 and 1.5 mg/ml matrine or 0.5, 1.0, 1.5, and 2.0 mg/ml oxymatrine. These quinolizidine alkaloids, found in liquorice confectionary and organic green tea are suspected to be aneugenic or clastogenic. The MicroFlow test showed statistically significant increases in micronuclei formation from 1.0 mg/ml on, albeit with slightly reduced relative survival, the metric for cytotoxicity recommended for this method at the time of the assay implementation. Complementary testing using microscopical evaluation did not support these results due to excessive cytotoxicity observed at matrine concentrations of 1.0 mg/ml and higher. Additionally, mechanistic studies using the ToxTracker ACE assay indicated that matrine is not a direct clastogen. Herein, we illustrate and discuss the importance of appropriate metrics for cell proliferation and cytotoxicity which should account for the toxicological properties of the test compounds. The aim is to assure consistency between OECD TG-compliant evaluation methods and to avoid misleading positive findings when evaluating the biological relevance of increased micronuclei formation accompanied by cytotoxicity.

**Supplementary Information:**

The online version contains supplementary material available at 10.1007/s00204-025-04271-1.

## Introduction

Identifying and characterising potentially genotoxic substances is one of the main objectives of regulators in several different legislations to protect consumer health, as exposure to genotoxic substances may lead to the formation of cancer or congenital disorders. It is regularly distinguished between two important genotoxic endpoints: gene mutation and chromosome damage. The latter can present as clastogenicity i.e., the property to induce structural chromosome damage and/or aneugenicity i.e., the property to induce numerical chromosome damage. Clastogenicity is caused by DNA strand breaks whereas aneugenicity leads to chromosome gain or loss. As various regulatory silos such as drug safety or pesticide residues require substances to be tested for their clastogenic and/or aneugenic potential, several in vitro tests such as the mouse lymphoma assay (MLA), chromosomal aberration assay (IVCA) or micronucleus test (MN) have been developed, validated and harmonised by test guidelines.

The current gold standard is the mammalian cell in vitro micronucleus (MN) tests according to OECD test guideline 487 (2023). Not only is the micronucleus test known to be robust and reproducible, but it is the only test that can detect and differentiate between numerical (aneugenicity) and structural (clastogenicity) chromosome damage. Given the dose-dependent nature of aneugenicity, this allows to support the existence of safe threshold values, which may strongly influence regulatory decision making. The in vitro MN test uses primary cells or cell lines. Human or mammalian peripheral blood lymphocytes (HPBL) as well as human derived cell lines such as TK6 or mammalian cell lines such as CHO, CHL or V79 are commonly used (Fenech 2007). The cells are exposed to the test substance, fixed and stained to allow for conventional microscopic evaluation for the presence of micronuclei. The ‘manual’ evaluation of a complete test, however, can be very time consuming and error prone. Depending on individual experience the analysis of one slide may take up to one hour. Taking into account three test conditions, including solvent and positive controls, a considerable amount of time is needed for microscopy. Given the ever-increasing number of substances to be tested, the demand for faster, high-throughput methods is growing. Beyond that, test automation offers additional opportunities for increased standardisation, reducing human error and risk of bias. A common alternative to the (automated) microscopic method is based on flow cytometry. Developed by Litron Laboratories (Avlasevich et al. 2006; Bryce et al. 2007, 2008) the MicroFlow assay has gained the reputation to be fast, reproducible and less subjective as compared to tests, that require manual counting. Although the MicroFlow may not yet have found widespread regulatory application despite its inclusion in OECD TG 487, it has gained popularity in the pharmaceutical industry, especially in drug development as a fast screening method for drug candidates (Nicolette et al. 2011).

According to OECD TG 487 (2023), an in vitro MN test is considered clearly positive if it meets the acceptability criteria of the guideline and fulfils the following three criteria: (I) a concentration-related increase in the occurrence of MN under at least one experimental condition; (II) a statistically significant increase in MN frequency for at least one test concentration when compared to the solvent control; and (III) values that are outside the 95th percentile of the historical control data (HCD) of the solvent controls. An additional criterion for positive tests commonly mentioned in literature is an increase in MN frequency ≥ threefold. Substances should be tested up to a maximum concentration of 2 mg/ml, 2 µl/ml or 10 mM (whichever is lowest). If cytotoxicity occurs, the highest tested concentration should exert 55 ± 5% cytotoxicity. It is important to evaluate positive test results carefully, if they occur only at the upper end of the cytotoxicity range, as a high cytotoxicity may lead to an artefactual increase in MN frequency and thus, to false-positive results (Honma 2011; OECD 2023).

Several methods have been developed to confirm replication and to assess the cytotoxic potential of test substances. OECD TG 487 recommends the cytokinesis-block proliferation index (CBPI) or replication index (RI), if cytochalasin B is used and the relative increase in cell counts (RICC) or relative population doubling (RPD) if not (see Methods section). These parameters should be determined during the main experiment, although preliminary tests may help to establish the concentration range of the main experiment. Non-microscopical evaluation techniques may require the use of other metrics to estimate replication and cytotoxicity. The MicroFlow protocol *Version 141030* used a relative survival metric based on the ratio of intact, viable nuclei to counting beads involving lysis of the cells prior to analysis. Cytotoxicity-only assays, such as those for membrane integrity, necrosis or apoptosis are not recommended as replacement for the above mentioned count-based parameters but may provide additional information.

Finally, a new methodology integrating (geno)toxicity endpoints with mechanistic information is currently being evaluated in the OECD test guideline programme. The ToxTracker ACE assay, based on reporter cell lines can detect DNA damage (clastogenicity/aneugenicity), oxidative stress, unfolded protein response as well as general cellular stress within the same sample (Hendriks et al. 2012, 2016). Such assays may be useful as first-line follow-up to a positive in vitro MN assay before initiating any in vivo studies in laboratory animals.

In our study, we examined the effect of different implementations of the OECD TG 487 to assess the clastogenic and/or aneugenic potential of matrine. Additionally, a ToxTracker ACE assay was commissioned to a contract research laboratory.

Matrine is a quinolizidine alkaloid naturally occurring in plants of the *Sophora* family along with related alkaloid species, such as its N-oxide oxymatrine. Due to the insecticidal properties of matrine, it is used as biopesticide in some parts of the world and has been proposed for biocidal wood treatment against termites (Mao and Henderson 2007). Matrine has recently been found as a contaminant in liquorice confectionary ((BfR) 2023), as well as green tea leaves ((RASFF) 2023), likely resulting from co-harvesting. In addition to insecticidal properties, anti-inflammatory and anti-cancer effects are discussed for matrine and oxymatrine (Abd-Alla et al. 2021; Lan et al. 2020; Li et al. 2020; You et al. 2020; Zhang et al. 2020), but adverse effects such as reproductive toxicity, hepatotoxicity or neurotoxicity have been reported as well (Gu et al. 2018; Lu et al. 2016; You et al. 2020). The genotoxic potential of matrine was investigated in previous studies, most of these, however, did not investigate pure matrine, but *Sophora* extracts. A positive in vitro chromosomal aberration test was reported for hot water extracts of *Sophora flavescens Aiton* by (Che et al. 2015) and positive results in in vivo chromosomal aberration and micronucleus tests have been observed with hot water extracts of *Sophora flavescens Aiton* by (Yin et al. 1991). The most recent study investigated > 98% pure matrine in the in vivo alkaline comet assay and despite being reported as negative by the authors, an increase in the DNA tail from 1.3 ± 0.2% in control tissue to 4.2 ± 0.3% was observed in liver cells. Furthermore, (Q)SAR predictions for MA/OM could not reliably rule-out bacterial mutagenicity (Fischer et al. 2024b) or the potential to cause chromosome damage (Fischer et al. 2024a), however, the absence of a detectable potential of matrine and oxymatrine to induce gene mutations in bacteria was previously demonstrated.

With the aim to clarify the potential of matrine to cause chromosome damage, we herein show the results of the in vitro MN test, the MicroFlow analysis, and a ToxTracker ACE assay all conducted with matrine and discuss the importance of applying appropriate replication/cytotoxicity metrics to avoid potentially misleading positive findings when evaluating the biological relevance of increased micronuclei formation accompanied by significant cytotoxicity.

As demonstrated here, understanding strengths, weaknesses and pitfalls of alternative clastogenicity assays is key to improve confidence in regulatory decision-making, in particular with an inconsistent database as for matrine. This case study also illustrates the utility of mechanistic assays in this context.

## Materials and methods

### Cell culture and growth medium

V79 cells obtained from the German Institute of Human Nutrition (DIfE) were used in this study and cultivated in DMEM, supplemented with 1% penicillin/streptomycin (pen/strep) and 10% fetal calf serum (FCS; Sigma-Aldrich). The cells were incubated in a humidified atmosphere of 5% (v/v) CO_2_ at 37 °C. Doubling time was 12–14 h, sub-cultures were established every 2–3 days when cells reached confluency.

### Test substance and formulation

Matrine (MA) and oxymatrine (OM) used for the MicroFlow assay were obtained from MedChemExpress, USA with a purity of ≥ 98.0% for batch ID 27619 (MA) and 25568 (OM). The test substances were dissolved at a concentration of 200 mg/ml in DMSO (Merck, Darmstadt, Germany, ≥ 99.8%) to produce master stock solution, kept stored at − 20 °C. This master stock was dissolved in DMEM (Dulbecco’s modified Eagle medium; PAN Biotech) containing 1% penicillin/stryptomycin and 10% fetal calf serum (FCS) to make fresh stock solutions on the day of the experiment. Medium without FCS was used for experiments with metabolic activation, the final S9 concentration per well reached 1%. Matrine used for the in vitro MN test with microscopic evaluation was obtained from PhytoLab, Germany, with a purity of 99.66% for batch 24641. Matrine was dissolved in DMEM on the day of the experiments to produce working concentrations of 0.1, 0.5, 0.75, 1.0, 1.25, 1.375, 1.5 mg/ml.

### Treatment of cell cultures

The culture medium was aspirated, cells were washed with PBS (phosphate buffered saline) and replaced with the working solutions of (oxy)matrine, the solvent control or the positive controls ethyl methyl sulfonate (EMS) and vinblastine (Vin).

Preliminary WST-1 assays were performed, to identify appropriate test concentrations of (oxy)matrine. Cells were seeded in 48-well plates and grown for 24 h, treated with the test solutions for another 24 h and incubated with WST-1. After 1 h, absorption at 450 nm was measured. DMSO concentrations did not exceed 1% (v/v). MA and OM concentrations of 0.5, 1.0, 1.25, 1.375, 1.5 mg/ml) and 0.5, 1.0, 1.5, 2.0 mg/ml, respectively, were selected for further testing in the MicroFlow, based on the preliminary WST-1 assays.

### Cell counts and evaluation of replication/cytotoxicity

Different methods were applied to determine cell replication and/or cytotoxicity in the MN tests, based on either cell or nuclei counts.

The MicroFlow protocol *Version 141030* that was used at the time of the experiments recommended to use the relative survival to measure cytotoxicity. The calculation is based on the ratio of intact nuclei to inert counting beads, measured by flow cytometry. The calculated ratio for the solvent control is defined as 100% relative survival, the nuclei-to-bead ratios of the individual samples are related to that ratio.

$$Rel.\, survival= \frac{Nuclei{-}to{-}Bead\, ratio\, of\, treated\, samples}{Nuclei{-}to{-}Bead\, ratio\, of\, solvent\, control\, samples}*100\%$$

Proliferation/Cytotoxicity was determined according to OECD TG 487, which recommends the Relative Increase in Cell Counts (RICC) or Relative Population Doubling (RPD) for MN tests performed without cytochalasin B. Here, the final number of cells were the respective cell counts after 24 h of treatment, whereas the initial number of seeded cells was taken as the starting number of cells i.e., 8000 in 48-well and 30,000 in 6-well. A reduction of RICC or RPD in treatment groups to values below 100% was interpreted as cytotoxicity.

$$RICC=\frac{{[Final\, number\, of\, cells]}_{T} - {[Starting\, number\, of\, cells]}_{T}}{{[Final\, number\, of\, cells]}_{C} - {[Starting\, number\, of\, cells]}_{C}} \times 100$$

$$\% Cytotoxicity = 100 \%-RICC$$

$$RPD=\frac{{[Number\, of\, population\, doublings]}_{T} }{{[Number\, of\, population\, doublings]}_{C} } \times 100$$

$$Number\, of\, population\, doublings=\frac{1}{{log}_{10}2} \times {log}_{10 }\frac{[Final\, number\, of\, cells]}{[Starting\, number\, of\, cell]}$$

$$\% Cytotoxicity = 100 \%-RPD$$

The MicroFlow assay was conducted in 48-well plates, whereas the MN test with microscopic evaluation of the slides was conducted in 6-well plates.

#### 48-Well

The culture medium was removed, cells were washed with PBS and 100 µl of trypsin was added. Incubation time was between 5 and 7 min, depending on cell density. Another 400 µl per well of DMEM were added, the well was rinsed with the suspension several times and subsequently transferred to tubes. The suspension was mixed with equal amounts of trypan blue, pipetted on counting slides and the number of viable cells were measured with an EVE (NanoEntek) cell counter.

#### 6-Well

The culture medium was removed, cells were washed with PBS and 100 µl of trypsin was added. Incubation time was 3 min. Another 900 µl per well of DMEM were added, the well was rinsed with the suspension several times and subsequently transferred to tubes. 50 µl of the cell suspension were mixed with 5 ml of CASYton (buffer) and the number of viable cells were measured with a CASY Cell counter and Analyzer (OMNI Life Sciences).

### Micronucleus assay with evaluation by flow cytometry

The MicroFlow was performed using the standard protocol for adherent cells (MicroFlow Protocol *Version 141030*) with all volumes adjusted from 24-well to 48-well plates. To reach the recommended confluency of ~ 80% at the start of the treatment, 8000 V79 cells were seeded per well and grown for 24 h prior to the 4 h (± S9) or 24 h treatment. No cytochalasin B was used, as the assay is based on the counting of nuclei, following a two-step staining and cell lyses. After sample preparation according to the MicroFlow protocol, the samples were measured with a BD Accuri C6 (Becton–Dickinson) flow cytometer, 20,000 events were counted per sample and results were normalized to the micronucleus frequency (‰ MN) as micronuclei per 1000 events. The assays were conducted in three independent experiments, on three different days (biological replicates). In each experiment, four wells were treated per solvent control, three per test substance concentration and two for each positive control. Relative survival was calculated as described above.

### Historical control data MicroFlow

Results of 19, 21 and 27 independent experiments were collected for the treatment conditions 4 h (± S9) or 24 h respectively. Outliers were excluded to calculate the 95% confidence interval (CI) and prediction intervals (PI) (Table 1).

**Table 1:** MicroFlow HCD of concurrent solvent control

Treatment	Solvent	n	‰ MN			
			Mean	SD	95% CI	95%PI
4 h – S9	DMSO	19	1.8	0.4	1.6–2.1	1.0–3.3
4 h + S9	DMSO	21	1.9	0.3	1.8–2.2	1.1–3.0
24 h – S9	DMSO	25	2.2	0.4	2.1–2.5	1.3–3.4

### Micronucleus assay with microscopic evaluation

Positive results in the MicroFlow assay were followed up by MN tests using microscopical evaluation. Cells were grown on square-shaped 10 mm cover-slips coated with alcian blue for better cell adhesion and placed in 6-well plates. 30,000 cells were seeded per well and after a 24 h growth phase treated for another 24 h without cytochalasin B. Duplicate slides were prepared and analysed for each test substance concentration and positive control; quadruplicates were prepared and analysed for solvent controls.

For cell fixing the cover-slips were transferred to another 6-well plate filled with ice cold phosphate buffered saline (PBS) in methanol (MeOH/PBS 90/10) for 10 min and subsequently dried and coded. Cells were sequentially stained with acridine orange (125 mg/l PBS) for 1.5 min and washed twice with PBS for 2 and 8 min, respectively. The slides were subsequently analysed using fluorescence microscopy (Leica DM6000 B) with a triple band filter (blue, green, red). One thousand (1000) cells per coded slide were manually counted and micronucleus frequency (MN/1000) was calculated.

### Statistical analysis

Three independent MicroFlow assays per test condition and three independent in vitro MN tests (24 h -S9) were performed. The results were pooled for each type of test and GraphPad Prism was used for statistical analysis. One-way-ANOVAs with Geisser-Greenhouse correction, followed by Dunnet`s multiple comparison was conducted for the MicroFLow, the MN test with microscopic analysis was evaluated with Mixed-Effects analysis with Geisser-Greenhouse correction, followed by Dunnet`s multiple comparison.

### ToxTracker ACE

The ToxTracker ACE assay was performed by Toxys in Oegstgeest, Netherlands.This assay uses six GFP-based (green-fluorescent-protein) mouse embryonic stem (mES) reporter cell lines which contain distinct fluorescent reporters to indicate the test substances ability to cause protein damage, oxidative stress or DNA damage. DNA damage is detected by the reporters Bscl2 and Rtkn, oxidative stress is detected by Srxn1 and Blvrb. Activation of p53 is detected by Btg2 and protein damage by Ddit3. The biological reactivity of the test substances is detected via activation of the respective cellular signalling pathway after incubation for 24 h. Additional DNA staining allows differentiation between clastogenic/aneugenic properties. The induced GFP expression is measured as fluorescence using a flow cytometer. Mean GFP fluorescence relative to the solvent control is used to calculate the fold increase. If a substance causes a concentration-related increase of > 1.5-fold without excessive cytotoxicity (< 75%) the response is considered positive (Hendriks et al. 2012, 2011, 2016). Both matrine and oxymatrine were tested in 5 concentrations up to 200 mg/ml or 160 mg/ml, respectively in three independent experiments.

## Results

### Pre-test cytotoxicity

**Fig. 1:**
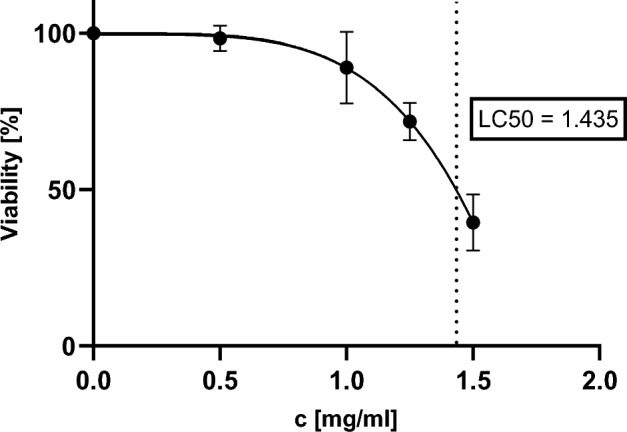
Cell viability in the WST-1 test after incubation of V79 cells with matrine for 24 h. Mean ± SD, n = 3

Matrine and oxymatrine were tested with WST-1 (water soluble tetracyclin), in order to determine the concentration range for the micronucleus assays. The 24 h incubation of V79 cells with matrine resulted in a relative viability of 39.4% at a concentration of 1.5 mg/ml and a LC_50_ = 1.435 mg/ml was calculated using non-linear regression. A maximum concentration of 1.5 mg/ml was chosen as the maximum concentration of matrine for the following tests. The 4 h ± S9 incubation with matrine and the 4 h ± S9 and 24 h incubation with oxymatrine did not decrease the cell viability (data not shown), therefore a maximum test concentration of 2.0 mg/ml was chosen (Fig. 1).

### MicroFlow

The MicroFlow assay was performed with matrine and oxymatrine for the conditions 4 h ± S9 and 24 h -S9. Results are shown as mean micronuclei per 1000 intact nuclei (‰ MN) for matrine in Tables 1, 2 and 3 while numerical data for oxymatrine are provided as supplementary material.

**Table 2:** MicroFlow assay: Matrine 4 h -S9

Conc. [mg/ml]	‰ MN: MicroFlow						
	Values	Mean	SD	RSD [%]	Fold increase	Rel. Surv. [%]	*p* values
0.0	2.4, 1.6, 1.6	1.9	0.5	24.5	1.0	100.0	–
0.5	2.0, 2.1, 1.8	2.0	0.1	5.8	1.1	102.9	0.9939
1.0	2.0, 2.3, 1.7	2.0	0.3	13.4	1.1	96.2	0.9903
1.5	2.2, 2.2, 1.7	2.0	0.3	13.5	1.1	91.7	0.9255
2.0	2.6, 2.5, 2.1	2.4	0.3	11.3	1.3	70.9	0.2924
EMS 0.5	5.6, 9.4, 7.5	7.5	1.9	25.7	4.3	74.1	0.1327
Vin 2.5 ng	11.3, 15.4, 8.6	11.8	3.4	28.9	6.5	85.7	0.1017

EMS = ethyl methyl sulfonate; Vin = vinblastine

**Table 3:** MicroFlow assay: Matrine 4 h + S9

Conc. [mg/ml]	‰ MN: MicroFlow						
	Values	Mean	SD	RSD [%]	Fold increase	Rel. Surv. [%]	*p* values
0.0	1.8, 2.2, 1.4	1.8	0.4	22.0	1.0	100.0	–
0.5	5.5, 6.4, 4.8	5.6	0.8	14.9	3.1	81.7	**0.0093**
1.0	5.1, 7.2, 4.6	5.6	1.4	24.8	3.1	76.9	0.0543
1.5	5.5, 7.1, 7.3	5.8	1.2	21.5	3.2	71.1	**0.0349**
2.0	7.9, 8.9, 7.3	8.0	0.8	10.0	4.6	48.8	**0.0036**
CP 2.5	47.7, 61.8, 61.3	56.9	8.0	14.0	32.9	42.2	**0.0169**

CP = cyclophosphamide

Treatment of the V79 cells with matrine for 4 h without metabolic activation did not lead to increased micronucleus formation (Table 2). A One-way-ANOVA with Geisser-Greenhouse correction, followed by Dunnett`s multiple comparison was performed, but did not indicate significance. The relative survival at the highest test concentration of 2 mg/ml matrine decreased to 70.9%, indicating slightly cytotoxic effects. The solvent control and the two positive controls were within the respective prediction intervals of our historical control data (HCD) and the positive controls showed a 4.3-fold (EMS) and 6.5-fold (Vin) increase.

Treatment of the V79 cells with matrine for 4 h with metabolic activation led to increased micronucleus formation (Table 3) starting at 0.5 mg/ml (3.1-fold). The One-way-ANOVA with Geisser-Greenhouse correction, followed by Dunnett`s multiple comparison was performed and indicated a positive trend and significant increases for 0.5, 1.5 and 2.0 mg/ml. The relative survival at the highest test concentration of 2 mg/ml matrine decreased to 48.9%, indicating elevated cytotoxic effects. The solvent control and the positive control were within the respective prediction intervals of our historical control data (HCD) and the positive controls showed a 33.9-fold (CP) increase.

**Fig. 2:**
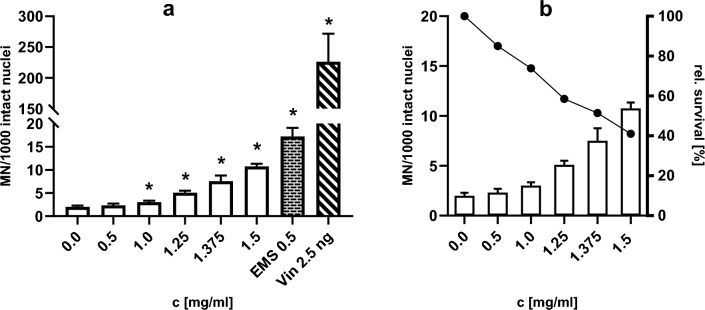
Results of the MicroFlow assay. **a** Micronuclei per 1000 intact nuclei after treatment of V79 with matrine, EMS or vinblastine for 24 h given as MEAN + SD, n = 3. Asterisks indicate the statistical significance of MN frequency relative to the solvent control (*p < 0.05, One-way-ANOVA). **b** Relative survival after treatment with matrine

Treatment of the V79 cells with matrine for 24 h led to increased micronuclei frequencies (Table 4, Fig. 2). One-way-ANOVA with Geisser-Greenhouse correction, followed by Dunnett`s multiple comparison indicated a strong positive trend and significant increases starting at a concentration of 1 mg/ml. At this concentration, the effect was moderate with a 1.5-fold increase and went up to 5.5-fold at 1.5 mg/ml. Concomitantly the relative survival decreased from 85% at a concentration of 0.5 mg/ml to 41% at 1.5 mg/ml, indicating elevated cytotoxicity. The solvent control was within the respective prediction intervals of our historical control data (HCD). The two positive controls were outside the prediction interval, but within the range of the HCD, showing a 8.7-fold (EMS) and 112.7-fold (Vin) increase. Of note, the relative standard deviation (RSD) calculated as percent of SD over Mean MN was 5.3%.

**Table 4:** MicroFlow assay: Matrine 24 h -S9

Conc. [mg/ml]	‰ MN: MicroFlow						
	Values	Mean	SD	RSD [%]	Fold increase	Rel. Surv. [%]	*p* values
0.0	1.7, 2.3, 2.0	2.0	0.3	15.1	1.0	100	–
0.5	2.4, 2.7, 1.9	2.3	0.4	16.6	1.2	85.0	0.6507
1.0	2.6, 3.2, 3.2	3.0	0.3	11.4	1.5	73.8	**0.0275**
1.25	4.6, 5.2, 5.4	5.1	0.4	7.9	2.6	58.5	**0.0082**
1.375	6.1, 8.4, 8.1	7.5	1.2	16.5	3.8	51.4	**0.0288**
1.5	10.4, 10.5, 11.4	10.8	0.6	5.3	5.5	41.0	**0.0044**
EMS 0.5	15.8, 19.3, 16.6	17.2	1.8	10.5	8.7	56.2	**0.0096**
Vin 2.5 ng	186.7, 276.1, 215.1	226.0	45.7	20.2	112.7	37.7	**0.0373**

EMS = ethyl methyl sulfonate; Vin = vinblastine

The treatment of V79 cells with oxymatrine for 4 h with and without metabolic activation did not lead to an observable trend or increased micronucleus formation. However, the 24 h treatment did lead to a slight increase, starting at an oxymatrine concentration of 1 mg/ml (see Tables 7–9 in supplementary data). One-way-ANOVA with Geisser-Greenhouse correction, followed by Dunnett`s multiple comparison indicated a positive trend and significant increases starting at a concentration of 1.5 mg/ml. At this concentration, the effect was very weak with a 1.3-fold increase that went up to 1.7-fold at 2.0 mg/ml. Relative survival decreased to 75% at 2.0 mg/ml. Both MN frequencies were within the PI of the solvent control of our HCD.

### Microscopic in vitro micronucleus test

As a follow-up to the positive results of the MicroFlow assay, the in vitro MN test with microscopic evaluation was performed analogously in V79 cells. Investigations focussed on the 24 h incubation with matrine, as the test condition with the clearest outcome. The results are summarized in Table 5. A maximum test concentration of 1 mg/ml matrine could be achieved, due to strong cytotoxic effects observed at concentrations of 1 mg/ml. Higher concentrations could not be analyzed due to lack of countable cells. Additional lower concentrations were tested instead. In the follow-up test the 24 h treatment of V79 cells with matrine did not lead to increased micronucleus formation up to the maximum test concentration. Mixed-effects analysis with Geisser-Greenhouse correction followed by Dunnett`s multiple comparison did neither indicate a trend or significance The positive controls EMS and vinblastine did lead to 4.7 and 9.1-fold increase in micronucleus formation. It can further be noted, that RSD were generally higher than for the MicroFlow method and in the range 8–76%.

**Table 5:** Micronucleus Test (Microscopic Analysis): Matrine 24 h -S9

Conc. [mg/ml]	‰MN: TG 487							
	Values	Mean	SD	RSD [%]	Fold increase	RICC [%]	RPD [%]	*p* values
0.0	15.1, 10.4, 9.1	11.5	3.2	27.5	1.0	100	100	–
0.1	21.8, 6.9, 12.0	13.6	7.6	55.7	1.2	93.7	97.5	0.9501
0.25	17.6, 7.9, 5.0	10.2	6.6	65.1	0.9	75.6	89.4	0.9489
0.5	–, 5.5, 6.9	6.2	3.6	58.8	0.5	48.5	73.4	0.4470
0.75	8.4, 9.0, 13.8	10.4	3.0	28.7	0.9	61.6	81.8	0.9979
1.0	9.3, 10.9, 10.6	10.3	0.8	8.2	0.9	37.2	64.3	0.9782
1.25	n.t	n.t	–		–	14.5	37.0	–
1.375	n.t	n.t	–		–	8.1	24.4	–
1.5	n.t	n.t	–		–	8.3	24.8	–
EMS 0.25	100.6, 25.8, 35.0	53.8	40.8	75.9	4.7	56.9	79.0	0.4733
Vin 1.25 ng	66.8, 136.2, 110.3	104.4	35.0	33.6	9.1	32.1	59.6	0.1391

EMS = ethyl methyl sulfonate; Vin = vinblastine; n.t. = not tested

### ToxTracker ACE

Matrine (Fig. 1, Table 6) did not activate the genotoxicity reporters Bscl2-GFP, Rtkn-GFP nor the p53 activation and apoptosis marker Btg2-GFP in the ToxTracker ACE assay using murine embryonic stem cell strain B4418 (mES), neither in the absence nor presence of S9. However, an unfolded protein response as indicated by Ddit3-GFP and an activation of the Srxn1-GFP oxidative stress reporter were triggered. In the absence of S9, the Srxn1-GFP response was weak in all three replicates and thus classified as “equivocal”. The twofold cut-off value was only exceeded at the highest tested concentration of 2000 µg/ml with a corresponding cytotoxicity of 75%.

For oxymatrine (Fig. 4, Table 6), a moderate activation of the oxidative stress response markers Srxn1-GFP and Blvrb-GFP was observed in the absence of S9, but the response remained below twofold at the highest tested concentration of 1600 µg/ml.

**Fig. 3:**
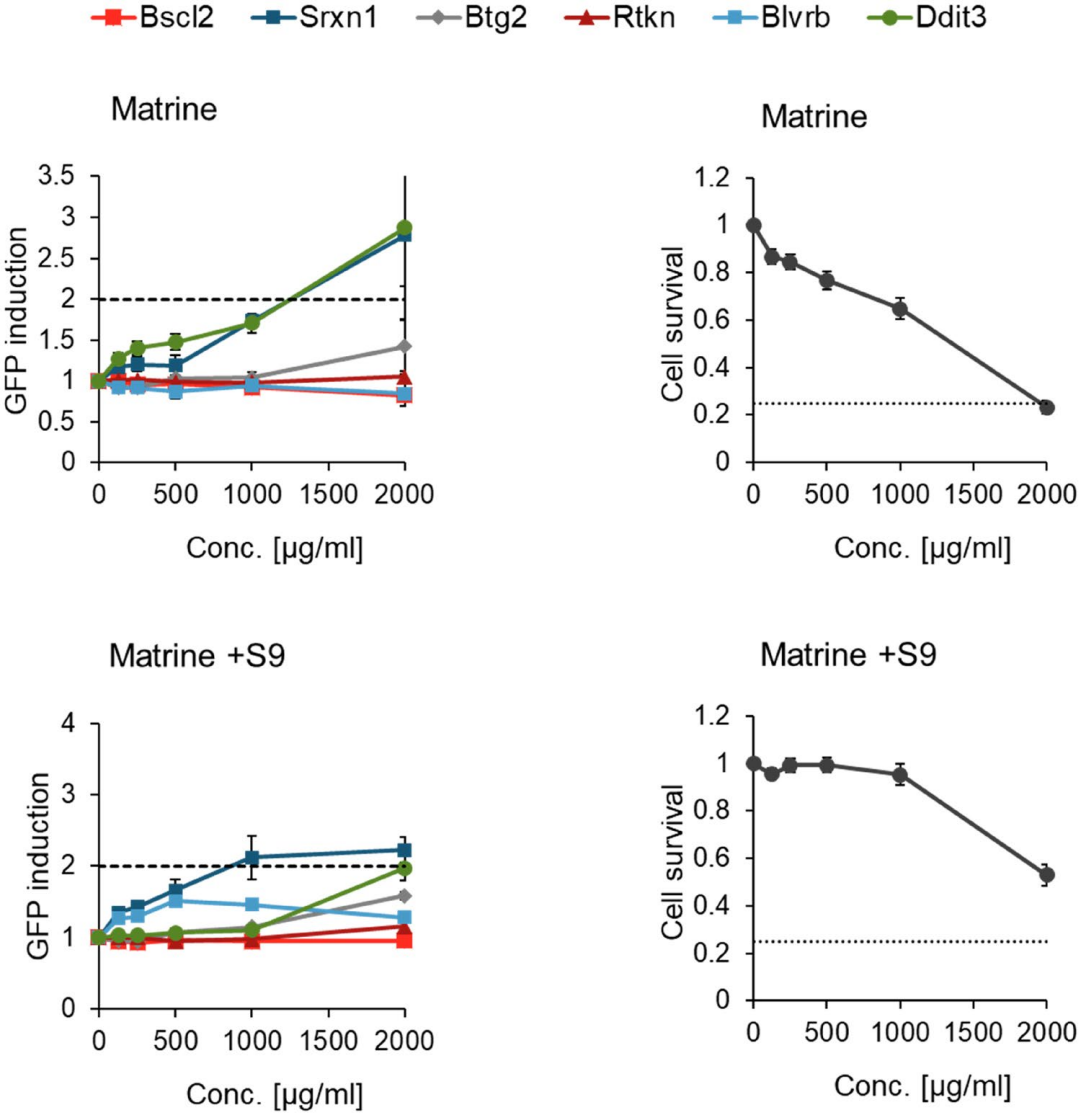
GFP induction for six marker genes and cell survival after 24 h incubation with matrine in absence and presence of 0.4% S9 mix, given as mean ± SEM, n = 3

**Fig. 4:**
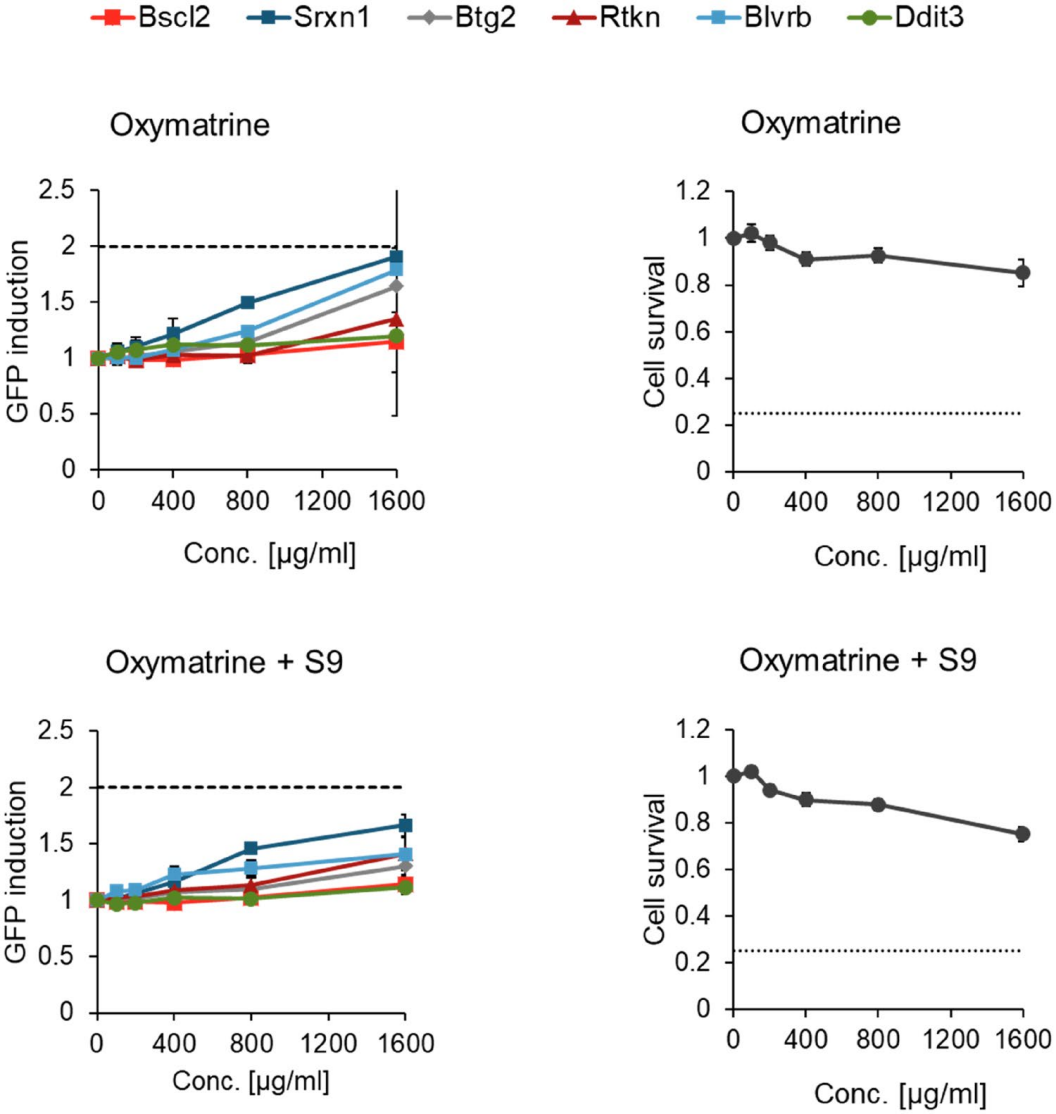
GFP induction for six marker genes and cell survival after 24 h incubation with oxymatrine in absence and presence of 0.4% S9 mix, given as mean ± SEM, n = 3

**Table 6:**
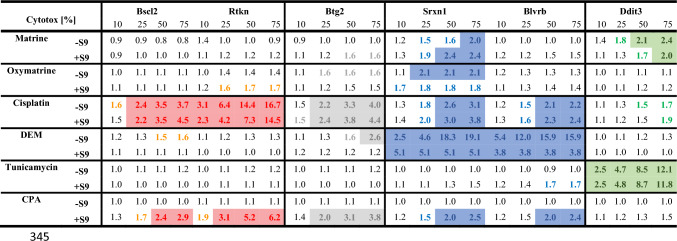
GFP induction: Matrine & Oxymatrine

## Discussion

### Matrine induces statistically increased micronucleus formation in the MicroFlow assay

The OECD TG 487 defines specific acceptability criteria for a valid in vitro MN test. Substances should be tested up to a concentration of 2 mg/ml, 2 µl/ml, 10 mM or until 55 ± 5% (i.e. 50–60%) cytotoxicity is achieved. Regarding concurrent controls MN frequencies should lie within the respective control ranges of the solvent control and positive control HCD and positive controls should induce statistically significant increases. As these criteria are met in the 24 h incubation experiment with matrine, the test is considered acceptable. Criteria for a clearly positive test are a statistically significant increase in at least one test concentration, a concentration-related increase (trend), and MN counts, that are outside the 95% confidence interval of the historical control data (HCD) of the solvent control. These criteria are met as well. The treatment of V79 cells with matrine for 24 h has repeatedly led to concentration-related increased micronucleus counts in the MicroFlow assay. A conducted one-way ANOVA of the pooled results showed statistically increased counts at concentrations of 1, 1.25, 1.375 and 1.5 mg/ml, a trend test showed a positive correlation between concentration and MN count. Furthermore, the MN frequencies starting at 1 mg/ml are outside the distribution of the 95% confidence interval (2.1–2.5) of the solvent control HCD. In our experiments the concurrently determined mean cytotoxicity, when expressed as 100%—relative survival (the standard proliferation metric of the MicroFlow assay at the time of the test performance), went up from 26.2% at 1 mg/ml to 59% at 1.5 mg/ml and thus does not exceed the 55 ± 5% range. As was shown by others, some substances only exhibit their clastogenic potential at the upper end of that range. Concluding on artificially positive results due to cytotoxicity does not seem justified in this context. In any case, excluding the result at 1.5 mg/ml due to cytotoxicity would not affect the test outcome, as 1.375 mg/ml still showed a significant (and ≥ threefold) increase of MN formation, well outside the 95% confidence interval at 48.6% cytotoxicity.

### The microscopic evaluation method did not confirm the positive result of the in vitro MicroFlow micronucleus test

Although sensitive high(er)-throughput methods are obvious choices for an initial screening, it is of utmost importance to assure accuracy and reproducibility of the overall testing strategy. False-negative results may lead to the authorization and commercialization of potentially harmful substances, whereas false-positive results can result in a (premature) exclusion of potentially promising lead candidates during drug discovery in pharmaceutical research or regrettable substitution of pesticidal active substance by less sustainable alternatives. Therefore, positive in vitro mutagenicity findings usually trigger follow-up in vivo tests for ultimate clarification. However, in vivo testing is not only more costly and time consuming, a high false-positive in vitro rate would also be challenging the 3-R principle by triggering unnecessary animal testing. Therefore, the experimental conditions leading to a positive result in the MicroFlow assay, i.e. incubation with 0.5, 1.0, 1.25, 1.375, 1.5 mg/ml matrine over 24 h were also examined using the microscopic evaluation method. Marked cytotoxicity of matrine, starting at concentrations of 1 mg/ml, interfered with the cell-growth and lead to detachment of cells after the treatment and during the cell fixation and/or subsequent staining. The metrics RICC and RPD were determined to quantify the cytotoxicity as recommended by the OECD TG 487. Additional lower concentrations of 0.1, 0.25, 0.5, 0.75 mg/ml were included for a better description of the dose–response and to have at least three analyzable concentrations. To our surprise, no significant treatment-related increase in MN formation was observed in this test. However, some limitations of the microscopic method potentially impacting its sensitivity must be noted. As previously mentioned, concentrations above 1 mg/ml matrine could not be analyzed microscopically due to loss and degeneration of treated cells, unlike for the MicroFlow method. Cell numbers evaluated microscopically are typically one order of magnitude lower than the number of events scored in flow cytometry. In combination with a higher variance – relative standard deviation was between 5.3 and 16.6% for the MicroFlow (24 h) and 8.2 to 56.1% for the microscopic method (24 h). This may generally limit the statistical power of the assay.

### Proliferation/cytotoxicity metrics

A multitude of metrics have been developed to assess potential cytotoxicity of test items, which, as mentioned earlier, is paramount when assessing the relevance of positive, as well as negative test results. Common methods determine cytotoxicity as decreased cell viability or membrane integrity and are based on, e.g. the cells capability to convert dyes into a fluorescent form (WST-1; mitochondrial enzyme activity) or the lysosomal uptake (NRU) thereof. Other methods determine cytotoxicity as the decrease in cell proliferation and are based on cell counts or the ratio of cells to, e.g., inert beads. Unsurprisingly values of different metrics may only be roughly comparable, due to the different underlying principles of these metrics or the assays they are used in. This can complicate the evaluation and comparison of test results gained from, in principle similar assays, such as the here described in vitro MN tests using flow cytometry or microscope analysis. The relative survival metric was applied to determine cytotoxicity in the MicroFlow assay as it was the sole recommended metric (*Version 141030)* at the time the assays were performed. The relative survival metric has the advantage, that it can be measured concurrently with sample analysis, as it is based on the ratio of intact nuclei to counting beads and does not require additional experimental steps other than the addition of the beads. In our experiment the relative survival decreased to 41% at 1.5 mg/ml matrine, however, the next lower concentrations of 1.375 mg/ml and 1.25 mg/ml only decreased the relative survival to 51.4% and 58.5% respectively. As all criteria for a positive test were met, it could have been regarded as positive, even if the result at the highest concentration would have been declared non-relevant, due to cytotoxic effects.

The OECD TG 487 recommends the RICC or RPD for MN tests performed without cytochalasin B, therefore both RICC and RPD were determined in our confirmatory experiment. Both metrics are based on cell counts before and after the treatment, i.e., cell proliferation. The excessive cytotoxicity of matrine became apparent by microscope analysis of the test slides during the confirmatory MN test, as concentrations of matrine above 1 mg/ml could not be analysed. In order to determine the RICC and RPD additional cell treatments with up to 1.5 mg/ml matrine were performed and cells subsequently counted. Both values generally indicated a stronger cytotoxic (cytostatic) effect of matrine after 24 h incubation than the relative survival. At a matrine concentration of 1 mg/ml the RICC decreased to 37.2%, corresponding to 62.8% cytotoxicity/cytostasis, dropping to 8.3%, corresponding to 91.7% cytotoxicity/cytostasis at 1.5 mg/ml matrine. The RPD for the same concentrations decreased from 64.3 to 24.8%, i.e. from 35.7 to 75.2% cytotoxicity/cytostasis. Consequently, the positive MicroFlow results in our first experiment would have to be regarded as not biologically relevant, based on the RICC values. Based on the RPD on the other hand, 1 mg/ml matrine, being the lowest concentration that still led to a statistically significant increase in MN frequency would not exceed the threshold of 55 ± 5% cytotoxicity/cytostasis and therefore might be regarded as relevant. Lorge et al. (2008) and Kirkland (2010) concluded that both RICC and RPD are suitable to measure cytotoxicity/cytostasis, although the RPD often indicated lower cytotoxic/cytostatic effects than the RICC in the evaluation by Kirkland. Furthermore Honma (2011) has demonstrated, that the RPD may underestimate cytotoxicity/cytostasis in cases of higher numbers of population doublings (PD), e.g. due to longer treatment durations or fast cell proliferation. Therefore, we therefore regard the RICC as the more descriptive metric in this experiment and thus conclude the positive results in the MicroFlow to be artefactual.

Our findings illustrate, that the relative survival metric in the MicroFlow assay may have led to the conclusion of matrine possessing clastogenic/aneugenic potential in vitro due to unequivocal underestimation of the cytotoxicity of matrine. This underestimation however, may be explained to some extent by the different experimental conditions, especially the seeding density of the cells. The MicroFlow assay requires 80% confluency prior to flow cytometry analysis, which is unpractical when microscopically analysing slides with attached cells. Different cell densities may result in different total cell counts (in the same format), which can result in more or less pronounced cytotoxicity of the same concentration of a given compound. It should be noted, that the revised protocol version 240319 of the in vitro MicroFlow assay also recommends the RICC and RPD to assess cytotoxicity/cytostasis, however, RICC and RPD values may still differ (Fig. 2).

### Mechanistic considerations

The ToxTracker ACE assay was performed for both matrine and oxymatrine, to gain further mechanistic insights and possible explanations for our findings. As described in the results section neither matrine (Fig. 3, Table 6) nor oxymatrine (Fig. 4, Table 6) caused a twofold induction of the genotoxicity reporters Bscl2-GFP and Rtkn-GFP, the threshold for a positive response in the ToxTracker assay. The reporter Bscl2-GFP is activated upon formation of bulky DNA lesions and subsequent DNA replication stress, whereas Rtkn-GFP is activated upon induction of DNA double strand breaks. Both markers are usually activated by directly DNA reactive substances, while activation of Rtkn-GFP only is often observed for indirectly genotoxic substances, causing oxidative damage or aneugenicity. The non-activation of the aforementioned reporters support the assumption that non-genotoxic effects have led to the positive results in the MicroFlow assay seen for oxymatrine (24 h) and in particular for matrine (24 h). Furthermore, oxymatrine caused an “equivocal” 1.9-fold induction of the cellular oxidative stress reporter Srxn1-GFP in the absence of metabolic activation, whereas matrine caused an “equivocal” 1.6-fold induction in the absence of metabolic activation at 50% cytotoxicity and a 2.4-fold induction in the presence of metabolic activation. These results may indicate moderate potential of MA/OM to cause oxidative stress and explain the observed positive results in the MicroFlow assay. Lastly, at 50% cytotoxicity matrine caused a 2.1-fold induction of the reporter Ddit3-GFP in the absence of metabolic activation and an “equivocal” 1.7-fold induction in the presence of metabolic activation. This reporter is associated with the unfolded protein response, as well as cell cycle arrest and finally apoptosis (Tabas and Ron 2011), which in turn ultimately leads to DNA breakage and chromosome fragmentation (Hetz et al. 2020). In conclusion, the ToxTracker assay did not indicate direct genotoxicity of matrine or oxymatrine, but the potential to cause oxidative stress. Additionally, matrine may cause protein damage, cell cycle arrest and apoptosis. Overall, this provides a plausible mechanistic explanation for the positive results for matrine in the in vitro MicroFlow assay at very high, cytotoxic concentrations and the significant, yet non-relevant results for oxymatrine.

### Weight-of-evidence and overall analysis

The in vitro MicroFlow assay was positive for matrine, showing a clear dose–response and statistically significant MN frequencies outside the 95% confidence interval of the HCD at concentrations of 1 mg/ml and higher. The maximum target cytotoxicity of 55 ± 5% was reached at 1.5 mg/ml according to the cytotoxicity metric of relative survival. These apparently positive results were not confirmed by additional MN tests as microscopic analysis. Notably, microscopic evaluation was technically not possible at concentrations above 1 mg/ml. Determination of the cytotoxicity/cytostasis metrics RICC and RPD revealed the strong cytotoxic/cytostatic properties of matrine and the underestimation thereof by using the relative survival metric. The results in the in vitro MicroFlow assay for oxymatrine after 24 h treatment also showed a noticable dose–response and significant MN frequencies at concentrations of 1.5 and 2.0 mg/ml, but, cytotoxicity did not exceed 25%. Here, only the highest concentration caused MN frequencies outside the the 95% confidence interval of our HCD.

It should be noted that the suitability of the 95% confidence interval as a control limit has been put into question (Dertinger et al. 2023; Kluxen et al. 2021) and the prediction interval is recommended instead (Menssen 2023). Keeping that in mind the MN frequency for OM at 2 mg/ml can be regarded as biologically not relevant, as they are within the range (1.3–3.4) of the PI of our solvent control HCD. In case of matrine, however, the MN frequencies above 1 mg/ml would still be outside the PI.

The mechanistic information provided by the ToxTracker assay, however, supports the lack of direct DNA-reactivity of matrine and oxymatrine and shows their potential to cause oxidative stress.Additionally it indicated the potential of matrine to cause cell cycle arrest and apoptosis. Taken together this information would have provided a mechanistic basis for the positive results of matrine and oxymatrine in the MicroFlow assay.

Overall, we therefore conclude matrine and oxymatrine as negative in the in vitro MN assay, and the outcome of the MicroFlow assay for matrine as irrelevant positive due to excessive cytotoxicity. This interpretationsis further supported by the negative results of additional in vitro MN studies performed at a contract research laboratory, in which matrine and oxymatrine were tested with human peripheral blood lymphocytes ((BfR) 2023). All in all the evidence against a clastogenic/aneugenic potential of matrine and oxymatrine in the in vitro MN test clearly outweighs the evidence for it.

## Conclusions

In our study we describe a genotoxicity testing strategy, that combined New-Approach-Methods, such as (Q)SAR predictions (data not shown), the in vitro MicroFlow assay and the ToxTracker assay with conventional OECD TG-compliant MN studies in order to increase the reliability of the evaluation of the clastogenic/aneugenic potential of matrine and oxymatrine. We highlight factors that may impact the outcome of genotoxicity assessments, exemplified by the described evaluation of the clastogenic/aneugenic potential of matrine. Based on our results,we see the potential of the MicroFlow assay not only for screening purposes, but as an alternative to the conventional MN test. An alternative that allows faster sample analysis and was highly repeatable in our experiments. This high repeatability is reflected by the narrow ranges of the 95% confidence intervals and prediction intervals of our HCD and the lower relative SDs of the MN frequencies as compared to the manual counting. This high repeatability may also allow to determine significance at lower effect levels, potentially increasing the sensitivity of the assay. During the course of our study, we identified some potential for improvement, as we saw how unsuitable cytotoxicity/cytostasis metrics may result in misleading positive (or negative) findings, even when all evaluation criteria for a positive test result are met. Our study also demonstrated, how the ToxTracker assay could provide valuable mechanistic information, that helped to explain our observations and supported the overall assessment of matrine. In order to meet the growing demand for genotoxicity assessments of chemical substances, while at the same time reducing or replacing animal testing without sacrificing human health protection, it is necessary to increase confidence in promising new approach methods. This is of particular importance as they may have the potential to complement or perspectively replace conventional assays. To increase confidence from a regulatory perspective, assuring consistency of the outcomes of OECD TG-compliant studies on one hand and new high-throughput methods, such as the in vitro MicroFlow on the other hand is paramount. Thus, we see the need to update OECD TG 487 with respect to flow-cytometry-based methods and clarification of testing conditions, evaluation criteria and the selection of appropriate control limits for the historical control data.

## Supplementary Information

Below is the link to the electronic supplementary material.

Supplementary file 1 (DOCX 20 KB)

## Data Availability

All data generated or analysed during this study are included in this published article [and its supplementary information files].
