# Supplementary material for: How the methodology determines the outcome of the in vitro micronucleus assay (OECD TG 487): a comparison of the MicroFlow and the microscopic evaluation approach highlights the impact of cytotoxicity/cytostasis metrics in V79 cells for matrine
Source: Arch Toxicol. 2026 Jan 14;100(4):1603–15. doi: 10.1007/s00204-025-04271-1 (PMC13043565; doi:10.1007/s00204-025-04271-1)
Supplement: Supplementary file 1 — (DOCX 20 KB) [file 204_2025_4271_MOESM1_ESM.docx]

|  | | **Table 7 MicroFlow Data: Oxymatrine 4h** | | | | |  |  |
| --- | --- | --- | --- | --- | --- | --- | --- | --- |
|  | **‰ MN: MicroFlow** | | | | |  |  |  |
| **Conc.**  **[mg/ml]** | **Values** | | **Mean** | **SD** | **RSD [%]** | **Fold Increase** | **Rel. Surv. [%]** | **p-values** |
| 0.0 | 1.7, 1.7, 1.7 | | 1.7 | 0.0 | 0 | 1.0 | 100.0 | - |
| 0.5 | 2.1, 2.0, 2.5 | | 2.2 | 0.2 | 10.6 | 1.3 | 104.2 | 0.2061 |
| 1.0 | 1.8, 1.7, 2.5 | | 2.0 | 0.5 | 23.1 | 1.2 | 106.3 | 0.7269 |
| 1.5 | 2.0, 1.9, 1.9 | | 1.9 | 0.1 | 3.7 | 1.1 | 104.6 | 0.0517 |
| 2.0 | 2.8, 1.5, 2.0 | | 2.1 | 0.6 | 30.8 | 1.2 | 99.8 | 0.7872 |
| EMS 0.5 | 6.1, 8.2, 6.9 | | 7.0 | 1.0 | 14.9 | 4.1 | 68.8 | **0.0334** |
| Vinblastin 2.5 ng | 10.8, 13.0, 9.5 | | 11.1 | 1.7 | 15.7 | 6.5 | 86.7 | **0.0304** |

|  | | **Table 8 MicroFlow Data: Oxymatrine 4h +S9** | | | | |  |  |
| --- | --- | --- | --- | --- | --- | --- | --- | --- |
|  | **‰ MN: MicroFlow** | | | | |  |  |  |
| **Conc.**  **[mg/ml]** | **Values** | | **Mean** | **SD** | **RSD [%]** | **Fold Increase** | **Rel. Surv. [%]** | **p-values** |
| 0.0 | 1.9, 2.4, 1.7 | | 2.0 | 0.3 | 17.3 | 1.0 | 100.0 | - |
| 0.5 | 2.1, 2.5, 1.6 | | 2.1 | 0.5 | 23.2 | 1.0 | 102.9 | 0.8891 |
| 1.0 | 2.3, 2.6, 1.8 | | 2.2 | 0.4 | 19.0 | 1.1 | 100.0 | 0.2712 |
| 1.5 | 2.2, 3.0, 2.2 | | 2.5 | 0.5 | 19.6 | 1.2 | 96.7 | 0.0816 |
| 2.0 | 2.1, 3.0, 2.1 | | 2.4 | 0.5 | 22.3 | 1.2 | 91.2 | 0.1746 |
| CP 2.5 µg | 25.1, 57.0, 61.6 | | 47.9 | 19.9 | 41.5 | 24.1 | 50.8 | 0.1354 |

|  | | **Table 9 MicroFlow Data: Oxymatrine 24h** | | | | |  |  |
| --- | --- | --- | --- | --- | --- | --- | --- | --- |
|  | **‰ MN: MicroFlow** | | | | |  |  |  |
| **Conc.**  **[mg/ml]** | **Values** | | **Mean** | **SD** | **RSD [%]** | **Fold Increase** | **Rel. Surv. [%]** | **p-values** |
| 0.0 | 2.3, 2.0, 1.6 | | 1.9 | 0.3 | 16.7 | 1.0 | 100.0 | - |
| 0.5 | 1.7, 1.7, 2.1 | | 1.9 | 0.2 | 12.7 | 1.0 | 106.7 | 0.9916 |
| 1.0 | 2.5, 2.4, 2.4 | | 2.4 | 0.1 | 2.8 | 1.3 | 99.5 | 0.2898 |
| 1.5 | 2.8, 2.6, 2.2 | | 2.5 | 0.4 | 13.8 | 1.3 | 89.3 | **0.0091** |
| 2.0 | 3.2, 3.4, 2.9 | | 3.2 | 0.3 | 8.5 | 1.7 | 75.1 | **0.0414** |
| EMS 0.5 | 16.2, 18.2, 12.4 | | 15.6 | 3.0 | 19.0 | 8.0 | 51.1 | **0.0339** |
| Vinblastin 2.5 ng | 277.5, 223.4, 243.4 | | 248.1 | 27.3 | 11.0 | 129.2 | 30.8 | **0.0106** |
